# Diagnostic accuracy of the Xpert MTB/RIF assay for tuberculous pericarditis: A protocol of systematic review and meta-analysis

**DOI:** 10.1371/journal.pone.0252109

**Published:** 2021-05-26

**Authors:** Guocan Yu, Fangming Zhong, Yanqin Shen, Hong Zheng

**Affiliations:** 1 Department of Thoracic Surgery, Zhejiang Chinese Medicine and Western Medicine Integrated Hospital, Zhejiang, China; 2 Zhejiang Tuberculosis Diagnosis and Treatment Center, Zhejiang Chinese Medicine and Western Medicine Integrated Hospital, Zhejiang, China; International Medical University, MALAYSIA

## Abstract

**Background:**

Tuberculous pericarditis (TBP) can lead to serious consequences. Early diagnosis and treatment are very important for TBP, but early diagnosis is still very challenging. This study aims to evaluate the diagnostic accuracy of Xpert MTB/RIF for TBP using meta-analysis method.

**Methods:**

We will search Embase, PubMed, the Cochrane Library, China National Knowledge Infrastructure (CNKI), and the Wanfang database for researches assessing the diagnostic accuracy of Xpert MTB/RIF for TBP until April 2021. Any types of study design with full text will be selected and included. The Quality Assessment of Diagnostic Accuracy Studies (QUADAS-2) tool will be used to assess the risk of bias. We will use version 15.0 of the STATA software with the *midas* command packages to carry out meta-analyses.

**Results:**

Evidence for diagnostic accuracy of Xpert MTB/RIF for TBP will be provided through the study, and this protocol will be submitted to a peer-reviewed journal for publication.

**Conclusion:**

This study will provide evidence of Xpert MTB/RIF for TBP.

## 1. Introduction

Tuberculosis (TB) is a major global public health threat to human health [1]. Tuberculosis-related mortality remains high in developing countries, especially among those co-infected with acquired immunodeficiency syndrome (AIDS) and tuberculosis [2]. *Mycobacterium tuberculosis* (MTB) can infect almost every part of the body, but the most common site of infection is the lungs, leading to pulmonary tuberculosis (PTB). Infections occurring outside the lungs are referred to as extrapulmonary tuberculosis (EPTB). Severe types of EPTB lead to increased tuberculosis-related mortality [3]. Tuberculous pericarditis (TBP) is a critical type of EPTB, with the human immune deficiency virus epidemic, the incidence of TBP has progressively increased [4]. TBP is the most common cause of pericarditis in areas with a high incidence of TB [4, 5]. In the absence of prompt and effective treatment, TBP can result in very serious consequences, such as pericardial tamponade, constrictive pericarditis, and even death [6]. TBP has a fatality rate of up to 17–40% at longer than six months [7]. To reduce the poor prognosis of TBP, early diagnosis and treatment are essential. However, the early diagnosis of TBP is still very difficult and is often postponed. The reason for this is that the amount of MTB in pericardial fluid is generally very low, which results in a low positive rate for the commonly used acid fast bacillus (AFB) smear, and MTB culture takes weeks to produce results and thus cannot guide early diagnosis. Other tests, such as pericardial effusion adenosine deaminase, although indirectly helpful in the diagnosis, do not provide a direct microbiological basis [8, 9].

Xpert MTB/RIF uses semi-nested real-time polymerase chain reaction to detect MTB DNA in specimens, with the ability to report MTB and rifampicin resistance results within two hours [10, 11]. Based on the good performance of Xpert MTB/RIF in the diagnosis of TB, the World Health Organization has recommended the test for the early diagnosis of TB since 2010. Xpert MTB/RIF is also applicable to EPTB, such as tuberculous meningitis and lymph node TB, and it has also shown excellent diagnostic efficacy [12]. The application of Xpert MTB/RIF in the diagnosis of TBP has its unique advantages. We will perform this systematic review and meta-analysis to synthesise evidence on the diagnostic accuracy of Xpert MTB/RIF for detection of TBP among people living in endemic areas.

## 2. Methods

### 2.1 Design and registration

This is a proposal to conduct a systematic review and meta-analysis of a diagnostic test accuracy to synthesise evidence on the diagnostic accuracy of Xpert MTB/RIF for detection of TBP. On the International Platform of Registered systematic Review and Meta-Analysis Protocols (INPLASY), we have registered the protocol with the registration number of INPLASY202060045 [13]. The proposed study will be performed following the Preferred Reporting Items for Systematic Reviews and Meta-Analysis for Diagnostic Test Accuracy (PRISMA-DTA) guideline [14].

### 2.2. Information sources

We will search the relevant studies in health-related databases such as Embase, PubMed, the Cochrane Library, China National Knowledge Infrastructure (CNKI), and the Wanfang database for researches, which assessing the diagnostic accuracy of Xpert MTB/RIF for TBP up to April 2021. We will also explore the references cited in reviews for possible researches.

### 2.3. Search strategy

Guocan Yu and Fangming Zhong will conduct the search strategies. We will be restricted to English and Chinese language in our search process. Guocan Yu will do study search using search strategies. Search strategy of PubMed will be listed as follows:

#1 “Pericarditis, Tuberculous” [Mesh] OR “Pericarditides, Tuberculous” OR “Tuberculous Pericarditides” OR “Tuberculous Pericarditis”
#2 “Tuberculosis” [Mesh] OR tuberculosis OR Tuberculoses OR “Kochs Disease” OR “Koch’s Disease” OR “Koch Disease” OR “*Mycobacterium tuberculosis* Infection” OR “Infection, *Mycobacterium tuberculosis*” OR “Infections, *Mycobacterium tuberculosis*” OR “*Mycobacterium tuberculosis* Infections”
#3 “Pericardial Effusion” [Mesh] OR “Effusion, Pericardial” OR “Effusions, Pericardial” OR “Pericardial Effusions” OR Hemopericardium OR Chylopericardium OR Chylopericardiums
#4 #2 AND #3
#5 “Extra pulmonary tuberculosis” OR “ Extrapulmonary tuberculosis”
#6 #1 OR #4 OR #5
#7 Xpert OR geneXpert
#8 #6 AND #7

The Cochrane Library, Embase, CNKI, and Wanfang databases will use the similar search formulae.

### 2.4. Eligibility criteria

#### 2.4.1. Type of studies

Any study design, if it had evaluated the accuracy of Xpert MTB/RIF for TBP. We will exclude case reports, articles written in languages other than Chinese and English, researches with < 10 specimens, conference reports, and abstracts without full articles.

#### 2.4.2. Participants

Participants living in TB endemic areas using Xpert MTB/RIF to diagnose TBP regardless of sex, age, and geographic locations.

#### 2.4.3. Index tests

We will consider Xpert MTB/RIF as index test.

#### 2.4.4. Comparator test

Comparator test (tests other than the reference standard) is not an obligatory criteria (single arm study can be enrolled if participants, intervention, outcomes are satisfied because this study will measure the diagnostic accuracy of Xpert MTB/RIF for TBP.

#### 2.4.5. Target conditions

Full-text original researches that assessed the Xpert MTB/RIF assay for TBP will be included. TBP is as defined by the authors in the primary studies. Clear and appropriate reference standards are defined in researches.

#### 2.4.6. Reference standards

A composite reference standard (CRS) or MTB culture will be defined as the reference standard in our study. Clinical symptoms, radiographic features, biochemical test results, smears, culture, histopathology, and response to anti-tuberculosis drugs constituted the reference standards in the CRS. Some or all of the factors with positive results will be considered positive for TBP. Cases will be considered as non-TBP if all the results are negative. We will use the CRS as defined in the original paper.

#### 2.4.7. Outcomes

The main outcome will be measured in terms of sensitivity and specificity of the index test. Sensitivity refers to the probability that the index test result will be positive in an infected case. Specificity refers to the probability that the index test result will be negative in a non-infected case [15, 16]. True positive (TP), false positive (FP), false negative (FN), and true negative (TN) values for the index test can be extracted or calculated directly from the studies.

### 2.5. Literature screening and selection

Primary search records will be imported into ENDNOTE X9.2 literature management software, according to eligibility criteria. Two investigators (Guocan Yu and Fangming Zhong) will independently assess the candidate articles by reviewing their titles and abstracts, followed by the full text, for inclusion. Discrepancies between the two investigators will be resolved by discussion with a third investigator (Hong Zheng).

### 2.6. Data extraction

We will extract data including first author name; publication year; country; TP, FP, FN, and TN values for the assay; cut-off value of the index test, reference standard; patient selection method; specimen type; some steps (e.g., homogenization); and condition along with other parameters. The same two investigators (Guocan Yu and Fangming Zhong) will independently extract the necessary information from each of the included articles; We will cross-check the data that we have obtained. Discrepancies in the two data sets will be settled by discussion with a third investigator, similar to the literature selection phase. Data from studies against two different reference standards will be treated separately.

### 2.7. Quality evaluation

Based on the two reference standards (CRS and culture), the two investigators will independently divide the studies into two groups and used a revised tool for Quality Assessment of Diagnostic Accuracy Studies (QUADAS-2) to assess study quality separately [17] and the discrepancy between the two investigators will be solved by discussion with a third investigator (Hong Zheng). QUADAS-2 comprises four domains: patient selection, index test, reference standard, and flow and timing. Each domain is assessed in terms of risk of bias, and the first three domains are also assessed in terms of concerns regarding applicability.

### 2.8. Data synthesis and statistical analysis

We will first obtain the values corresponding to TP, FP, FN, and TN in each included study, and calculated the estimated pooled sensitivity and specificity of the Xpert MTB/RIF associated with the 95% confidence interval (CI), against CRS or culture, using bivariate random-effects models. Forest plots for sensitivity and specificity will be generated for each study. The areas under summary receiver operating characteristic (SROC) curves (AUC) will be subsequently calculated. Plots observed data in ROC plane for visual assessment of threshold effect. *I*^2^ statistics will be used to assess heterogeneity between the studies and a reference standard. While 0% will indicate no observed heterogeneity, values greater than 50% will be considered to imply substantial heterogeneity [18]. We will explore different types of samples, different patient selection methods, decontamination methods, sample conditions, and homogenization as potential sources of heterogeneity, using subgroup and meta-regression analyses. Sensitivity analyses will be used to reanalyses studies without poor quality in terms of QUADAS-2 to check the robustness of analyses. At least four published studies will be required to perform the meta-analysis for predefined variable types. Data from studies against CRS and culture will be analyzed separately. According to the PRISMA-DTA statement, systematic review and meta-analysis of diagnostic test accuracy studies is not required to assess publication bias [14]. Stata version 15.0 (Stata Corp., College Station, TX, USA) with the *midas* command packages will be used to generate forest plots of sensitivity and specificity with 95% CI for each study and carry out meta-analyses and meta-regression analyses. The Grading of Recommendations Assessment, Development and Evaluation (GRADE) guideline will be used to assess the strength of the body of evidence [19]. The quality of evidence will be classified into 4 levels: high, moderate, low, and very low, and the strength of the recommendation will be graded as strong or weak.

## 3. Discussion

TBP is a serious form of EPTB, which can lead to very serious consequences. Early diagnosis and treatment are essential for the recovery. Xpert MTB/RIF is highly valued in the early diagnosis of TB and it allows the establishment of the diagnosis of TB much earlier. As far as we know, high quality diagnostic meta-analysis for the diagnostic efficacy of Xpert MTB/RIF in TBP is lacking. We hope that the results of this study will be helpful in the diagnosis of TBP and thus reduce the adverse consequences associated with TBP.

## Supporting information

S1 Checklist Preferred Reporting Items for Systematic review and Meta-Analysis Protocols (PRISMA-P checklist). (DOC)
